# Strategies for Differentiation of Corneal Endothelial Cells From Stem Cells: Current Advances and Challenges

**DOI:** 10.1155/sci/5329168

**Published:** 2026-10-08

**Authors:** Ling Li, Yan Huang, Yuanyuan Wu, Qiuping Wang, Jing Ji, Lizhen Wang

**Affiliations:** ^1^ Key Laboratory of Biomechanics and Mechanobiology Ministry of Education; Key Laboratory of Innovation and Transformation of Advanced Medical Devices Ministry of Industry and Information Technology; National Medical Innovation Platform for Industry-Education Integration in Advanced Medical Devices (Interdiscipline of Medicine and Engineering); Beijing Advanced Innovation Center for Biomedical Engineering School of Biological Science and Medical Engineering Beihang University Beijing 100191 China buaa.edu.cn; ^2^ Innovation Center for Medical Engineering and Engineering Medicine Hangzhou International Innovation Institute Beihang University Hangzhou 311115 China buaa.edu.cn

**Keywords:** cell-based therapy, cocultures, corneal endothelial cells, differentiation, mesenchymal stem cells, pluripotent stem cells

## Abstract

The corneal endothelium, located in the posterior layer of the cornea, is critical to the maintenance of corneal clarity. Due to the limited proliferative capacity of the corneal endothelium, cell loss or damage can affect visual health. The lack of healthy corneal donors worldwide limits corneal transplantation, which is still the most successful clinical treatment. Since stem cells possess self‐renewal capacity and can differentiate into various cell types including corneal endothelial cells (CECs), they represent a promising candidate for regenerative medicine. Many methods have been developed to induce stem cells to differentiate into CECs, mainly including chemical induction, physical induction, and coculture with adult corneal cells such as lens epithelial cells and CECs. In this review, the structure, function and marker identification of CECs are firstly introduced. Then, the methods and research progress regarding the differentiation of multipotent stem cells (mesenchymal stem cells [MSCs]) and pluripotent stem cells (embryonic stem cells [ESCs] and induced pluripotent stem cells [iPSCs]) into CECs are reviewed in detail, and both in vivo and clinical applications of stem cell‐derived CECs are discussed. Finally, the challenges in stem cell differentiation into CECs are summarized, and future development directions are suggested.

## 1. Introduction

Corneal endothelial cells (CECs) play an essential role in sustaining corneal transparency and preserving normal visual function [1, 2]. Corneal endothelial dysfunction can be triggered by endothelial diseases, trauma, infection, or endothelial cell loss after corneal transplantation. This disorder may result in corneal edema, opacification, and even irreversible blindness, representing a global clinical challenge [3, 4]. To date, corneal transplantation is still the leading therapy for severe corneal endothelial disease [5]. The management of corneal endothelial dysfunction has shifted away from full‐thickness corneal transplantation toward targeted replacement of pathological layers, as exemplified by Descemet’s membrane endothelial keratoplasty (DMEK) and Descemet’s stripping automated endothelial keratoplasty (DSAEK). These surgical modalities have decreased the risk of post‐transplant allograft rejection and maintained ocular anatomical integrity [2, 6, 7]. However, nearly all such keratoplasty techniques require high‐quality corneal endothelial donors, which are in short supply [2, 8]. Meanwhile, corneal transplantation related to corneal endothelial diseases is increasing [9]. Accordingly, it is of vital importance to explore alternative sources of CECs.

Unlike other terminally differentiated cells, CECs in vivo have limited proliferative capacity and stagnate at the G1 phase in the growth cycle [10]. One therapeutic strategy targets residual endothelial cells, whereby topical interventions enhance their migratory, adhesive, and proliferative potential [11]. Previous studies have explored multiple growth factors, including epidermal growth factor (EGF), platelet‐derived growth factor (PDGF), transforming growth factor β (TGF‐β, which inhibits AKT activation), fibroblast growth factor (FGF), as well as a Rho‐associated protein kinase (ROCK) inhibitor [12–15]. Studies have shown that intracameral injection of CECs combined with the ROCK inhibitor Y‐27632 in patients with bullous keratopathy can effectively elevate endothelial cell density [16]. In vivo data demonstrated that Y‐27632 delays the progression of endothelial degeneration and supports the recovery of CEC density after endothelial damage [11, 17]. However, strategies to enhance endogenous endogenous CEC proliferation or function still demand long‐term research owing to insufficient data regarding the safety and sustained efficacy of such topical compounds in treating corneal diseases [11].

Concurrently, therapeutic strategies that aim to add new, functionally competent cells to the depopulated endothelium cell layer are being developed [11]. Cultivating corneal endothelial cells in vitro represents a potential approach to ease the scarcity of donor corneas. Nevertheless, CECs obtained by in vitro culture exhibit lower cell density compared with the original donor corneas [18]. Hence, many research groups seek to evaluate the potential of stem cell‐derived CECs as an alternative cellular resource [2]. Possessing prominent proliferative ability, low immunogenicity, and the potential to develop into any tissue type of the organism, stem cells stand out as the favored cell source for such therapeutic strategies [19, 20]. The candidate stem cell sources cover corneal stromal stem cells, corneal limbal stem cells, embryonic stem cells (ESCs), various adult stem cells, and induced pluripotent stem cells (iPSCs) [21–24]. Stem cells have broad applications and a bright future in corneal tissue engineering [25]. However, a wide variety of protocols can be adopted to initiate cell differentiation. To date, there are no standard differentiation protocols available. Direct corneal endothelial transplantation using the differentiation capacity of stem cells suffers from an unrestricted differentiation spectrum [26]. Despite these challenges, stem cell differentiation into CECs still provides promising new strategies for the treatment of corneal diseases. By generating functional CECs for transplantation, it is feasible to restore patients’ vision and further advance the fields of regenerative medicine and corneal tissue engineering.

Although several excellent reviews have summarized the effects of stem cell differentiation into corneal‐related cells, most of these articles focus on a single type of stem cell or on the differentiation into multiple types of corneal cells, with insufficient attention to recent clinical breakthroughs and translational strategies [8, 27]. In this review, we firstly introduce the structure, function, and markers of CECs. Then, we discuss the methods and research progress regarding corneal endothelial differentiation of stem cells, including multipotent stem cells (mesenchymal stem cells [MSCs]) and pluripotent stem cells (ESCs and iPSCs), in detail, as well as the in vivo and clinical applications of stem cell‐derived CECs. Ultimately, we discuss the development trends and challenges in stem cell differentiation into CECs. This review synthesizes the latest advances, including the first‐in‐human clinical trial of HLA‐matched iPSCs‐derived CECs and cell purification technology [28, 29]; systematically compares different stem cell sources based on four key translational indicators (differentiation efficiency, purity, induction time, and safety); elaborates on the current translational bottlenecks and potential solutions; and highlights cutting‐edge strategies such as gene‐edited universal CECs. This review summarizes recent advances and existing challenges regarding the therapeutic application of induced stem cells for corneal endothelial dysfunction.

## 2. Corneal Endothelial Cells

### 2.1. Corneal Endothelial Structure and Function

The cornea is responsible for almost two‐thirds of the ocular total refractive capacity (Figure 1A) [32]. Occupying the anterior one‐sixth of the ocular wall, the cornea features no blood vessels and is composed of transparent connective tissue; its central segment measures ~0.5 mm in thickness, whereas the peripheral part reaches 1.0 mm [33, 34]. Structurally, the human cornea is composed of 5 recognized layers, including the epithelium layer, Bowman’s membrane, the stromal layer, Descemet’s membrane, and the endothelial layer (Figure 1B) [10, 35]. The corneal endothelium constitutes the innermost cellular layer of the cornea. Normally, in adult humans, the corneal endothelial monolayer has an average of 3000 cells/mm^2^ [19, 36]. They are polygonal, mostly hexagonal cells originated from the periocular neural crest‐derived mesenchymal cells [37, 38]. CECs form a thin, compact monolayer and preserve corneal transparency via two physiological mechanisms: they serve as a physical barrier to block aqueous humor infiltration into the cornea and actively pump redundant fluid out of corneal tissue (Figure 1C) [3, 39, 40]. Due to the stagnant state of CECs in the G1 phase in vivo and their limited regenerative capacity, cell enlargement and migration constitute the primary mechanisms underlying monolayer repair, and cell density gradually declines with age [2, 10, 41, 42]. Multiple factors, such as systemic influences, glaucoma, ocular inflammation, bullous keratopathy, Fuchs’ endothelial dystrophy, intraocular surgery, or eye trauma, can lead to irreversible corneal endothelial dysfunction [2, 4]. If the cell density falls under the threshold of 400–500 cells/mm^2^, normal corneal deturgescence regulation will be disrupted, followed by corneal edema and gradual corneal opacity that impairs vision permanently (Figure 1D) [9, 43, 44].

**Figure 1:**
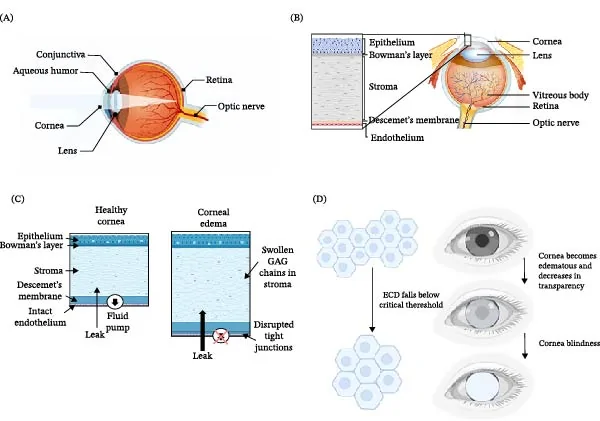
The structure of the cornea and the function of the corneal endothelium. (A) The human cornea contributes over 2/3 of the eye’s overall refractive power. Created with BioRender.com. (B) Cross‐sectional schematic of human eye with magnified corneal view [30]. (C) Diagram of corneas with normal and injured endothelium. Endothelial cells expel surplus fluid to maintain normal thickness in the healthy left cornea. Broken tight junctions and defective fluid pumps (red crosses) trigger fluid accumulation and corneal edema in the right specimen [31]. (D) Diagram showing the correlation between corneal endothelial cell density and corneal opacity [8].

### 2.2. The Marker Identification of CECs

Currently, corneal endothelial cell therapy has lately arisen as a highly promising therapeutic modality for the management of corneal endothelial dysfunction [4]. Reliable genotypic and phenotypic biomarkers serve as indispensable foundations if this cellular therapy is to be widely implemented in clinical practice (Figure 2) [45].

Figure 2The characteristics and identification of CECs. (A) Ranking of reported genes for identifying cultured human CECs (left). The widespread utilization of phenotypic markers reported over the past 10‐year period (right) [45]. (B) Induction of CECs from PBMC‐derived human iPSCs. Phase contrast photos at different culture stages reveal immature CECs at D6, and mature hexagonal CECs at D13 and D20. Note: Images with 20× and 40× magnification contain 50 μm scale bars [46]. (C) Quantitative RT‐PCR analysis and Gel image of RT‐PCR showing MSCs and CECs‐related gene expression.  ^∗^ *p* < 0.05 [47]. (D) Representative P0‐passage CEC cultures displaying ZO‐1, Na^+^/K^+^‐ATPase, aquaporin and N‐cadherin marker expression. Scale bar: 100 μm [18]. (E) WB analysis and protein levels of Na^+^/K^+^ ATPase and ZO‐1 in CECs compared to SKPs. Results are mean ± SEM.  ^∗^ *p* < 0.05 compared to SKPs [48]. (F) Flow cytometric detection was performed on revived B4G12 cells and iPSC‐originated CECs from Ff‐MH09s01, Ff‐I01s04, QHJI01s04. More than 90% of thawed CECs expressed CDH2, ATP1A1 and NR3C2, consistent with B4G12 endothelial cells [49].

**Figure:**
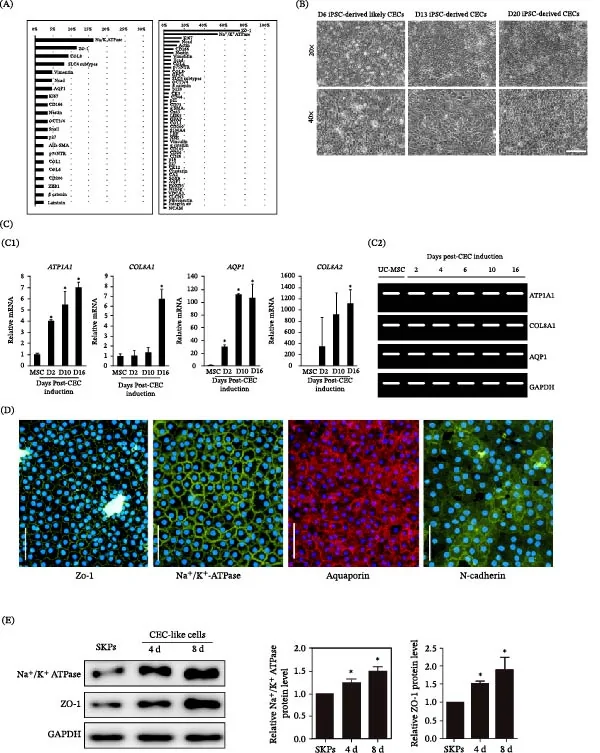

**Figure:**
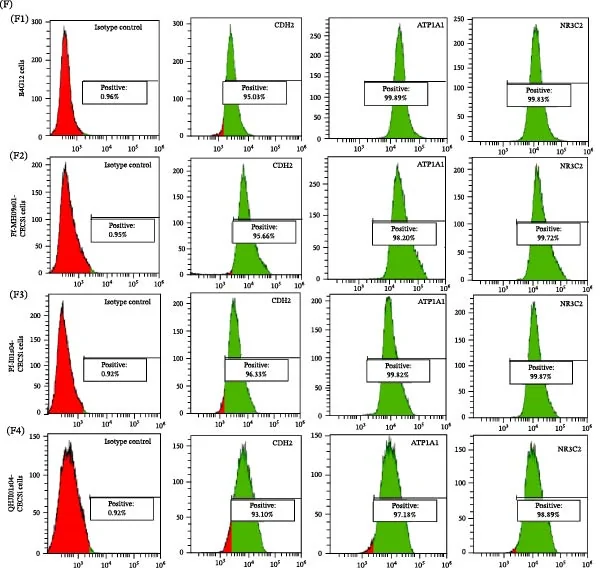

Accumulated studies have verified that CECs abundantly express sodium‐potassium adenosine triphosphatase (Na^+^/K^+^‐ATPase) and carbonic anhydrase [50]. The most vital physiological function of these cells is to transport ions within the corneal stroma, thereby driving excess fluid inside the cornea to flow outward. The Na^+^/K^+^‐ATPase and aquaporin 1 (AQP1) work in coordination to maintain the hydration homeostasis of corneal tissue [51, 52]. The Na^+^‐HCO_3_^-^ cotransporter is also indispensable as it mediates the transport of sodium ions and bicarbonate ions from the corneal stroma to endothelial cells [53, 54]. Sodium bicarbonate transporter‐like protein 11 (SLC4A11) exhibits high expression within the corneal endothelium and acts as a functional transporter in CECs; mutations in this gene lead to corneal endothelial disorders [55, 56]. Solute carrier family 4, sodium bicarbonate cotransporter, member 4 (SLC4A4), has also been implicated in corneal endothelial diseases in humans. Therefore, Na^+^/K^+^‐ATPase alpha 1‐subunit (ATP1A1), carbonic anhydrase type 2 (Car2), AQP1, SLC4A4, and SLC4A11 are usually selected as corneal endothelium markers. Tight Junction Protein 1 (TJP1, also known as zonula occludens‐1, ZO‐1) plays a role in signal transduction at cell–cell junctions and contributes to the formation of a selective barrier that regulates corneal hydration [57, 58]. It is reported that, among various CECs markers, the transcriptional abundance of ZO‐1 mRNA is markedly lower than the expression level of ZO‐1 protein [59, 60]. ZO‐1 and Na^+^/K^+^‐ATPase localize at the apical membrane of corneal endothelial monolayers. Their coexpression suggests a coordinated and dynamic regulation mechanism that maintains corneal transparency and homeostasis [61]. N‐cadherin (Cadherin 2 and CDH2) is highly expressed in the corneal endothelium. The main function of the N‐cadherin‐dependent apical junctional complex serves to selectively limit the permeation of water and solutes across paracellular gaps, while retaining the polarized distribution of ion channels such as Na^+^/K^+^‐ATPase [62]. Both ZO‐1 and N‐cadherin play vital roles in preserving intercellular junctions as well as retaining their hexagonal shape [63]. In addition, CECs express several other markers. For example, collagen type VIII alpha 1 (COL8A1) and alpha 2 (COL8A2) are highly expressed in CECs, participate in the construction of Descemet’s membrane, and thereby maintain the structural stability of corneal endothelium [64]. Collagen type IV alpha 2 (COL4A2), similar to COL8A2, is specifically expressed on the corneal endothelium [50].

Currently, there is a shortage of endothelial‐specific biomarkers, which hinders the identification of intact endothelial phenotypes in clinically transplantable grafts [65]. The lack of existing specific biomarkers creates a promising avenue to identify new markers for CECs. Yoshihara et al. [66] utilized single‐molecule sequencing for cap analysis of gene expression analysis and identified that POU6F2 was a vital regulator driving terminal differentiation in CECs. Ding et al. [67] conducted immunofluorescent staining on frozen tissue sections and characterized the antigen targets of two self‐generated monoclonal antibodies for the first time: TAG‐1A3 recognizes CD166/ALCAM; TAG‐2A12 binds membrane‐positioned peroxiredoxin‐6 (Prdx‐6), which is highly expressed on the plasma membrane of CECs [68], and this protein selectively binds the surface of CECs rather than other tested cell populations [67]. The paired‐like homeodomain transcription factor Pitx2 plays an essential role in the migration of cranial neural crest cells (NCCs) out of the neural tube and their subsequent differentiation into periocular mesenchyme (POM) [69]. Pitx2 together with Foxc1 serves as the signature for POM cells, whereas AP2α and Sox10 are classic markers to recognize NCCs [70]. By profiling mRNA transcriptomes, Chen et al. [71] demonstrated the high expression of Wnt5a, S100A4, S100A6, and IER3 in human fetal and adult corneal endothelium. Bartakova et al. [72] further combined microarray profiling and flow cytometry screening and discovered four surface markers (CD56, CD248, CAR, and CD109) capable of distinguishing intact CECs from cells undergoing endothelial‐mesenchymal transition (EMT). Notably, two canonical vascular endothelial markers, vWF and PECAM (CD31), cannot be detected in healthy CECs under physiological conditions [73, 74].

Overall, identifying key regulators of CECs phenotypes helps evaluate stem cell‐derived CECs. Full characterization of such CECs demands multiple analytical methods, including morphological evaluation, real‐time reverse transcription‐polymerase chain reaction (RT‐PCR, immunohistochemistry, flow cytometry, and karyotype analysis (Figure 2).

## 3. Induction of Stem Cells Into CECs With Coculture Methods

In vitro studies have verified that stem cells can be differentiated into CECs via three major induction strategies: coculture methods, biochemical factors, and mechanical stimuli. As a widely adopted induction approach, coculture refers to the simultaneous incubation of multiple cell populations within one culture environment, which is further categorized into direct and indirect contact modes. Direct coculture enables intimate intercellular contact yet complicates subsequent cell isolation. By contrast, indirect coculture physically separates distinct cell populations through barrier devices. Transwell inserts, as well as diverse natural and synthetic membrane materials, are the most commonly used separators in such systems [75]. Here, we summarized two indirect coculture methods for inducing stem cells to differentiate into CECs: the Transwell method and the conditioned medium method.

### 3.1. Indirect Contact Coculture Method Using the Transwell System

The Transwell coculture system consists of an upper cell insert and a lower culture well. A microporous membrane at the base of the insert blocks direct intercellular contact, yet permits free diffusion of molecular signals and soluble secretions derived from CECs [76].

Coculture systems are frequently employed for the directed differentiation of stem cells toward a specific lineage. MSCs, a type of adult stem cell featuring self‐renewal and multilineage differentiation potential, can be isolated from a wide range of human tissues including bone marrow, adipose tissue, dental pulp, skin, umbilical cord, and amniotic fluid. The easy accessibility of MSCs renders them a promising cell source for laboratory research and clinical translation [63, 77]. As multipotent stromal cells, MSCs hold the capacity to differentiate into multiple cell subtypes such as CECs [22, 78]. Coculture systems are also applied in the directed differentiation of pluripotent stem cells (ESCs and iPSCs) [79]. Although obtaining PSCs requires complex culture protocols and high‐cost culture reagents, PSCs present unique superiorities in differentiation potential, possess unlimited self‐renewal capacity, and have the potential to differentiate into all human somatic cell types [80, 81]. In vitro, PSCs can spontaneously form self‐assembled multizone ocular organoids, which recapitulate complete human eye development covering the ocular surface ectoderm, lens, neuroretina, and retinal pigment epithelium [82, 83]. During eye development, CECs are derived from the cranial NCCs, particularly POM [37, 84]. Accordingly, the majority of current in vitro protocols for generating CECs from ESCs or iPSCs are designed to recapitulate native ocular developmental cascades [85]. In the first step, NCCs are derived from ESCs/iPSCs. Next, CECs are derived from NCCs [86, 87].

In the Transwell system, signal cells including B4G12, corneal stromal cells, and lens epithelial cells can induce stem cells to differentiate into CECs. For example, Shao et al. [76] cocultured human fetal bone marrow‐derived endothelial progenitor cells (EPCs) with CECs in a Transwell system for 10 days, resulting in the expression of corneal endothelial cell‐specific markers AQP1 and neuron‐specific enolase (NSE). Transplantation of differentiated cells carried by porcine acellular corneal matrices into endothelium‐stripped cat corneas successfully recovered corneal transparency at day 28. Shen et al. [48] cocultured human skin‐derived precursor cells (SKPs) and B4G12 in a Transwell system. SKPs are neural crest‐derived embryonic precursor cells and share biological traits with neural crest stem cells (NCSCs). B4G12 is an immortalized human CECs line. Cocultivation of the two cell types confirmed that SKPs can differentiate into CEC‐like cells, which displayed morphology and biological characteristics analogous to primary human CECs. Furthermore, Shen et al. [88] investigated the possible differentiation molecular mechanism, including phosphatidylinositol 3‐kinase/protein kinase B (PI3K/Akt) pathways, mitogen‐activated protein kinase/extracellular‐signal‐regulated kinase (MAPK/ERK) pathways, Wnt/β‐catenin pathways, and transcription factors Pitx2 and Foxc1. The induced cells exhibited favorable adhesion and proliferation on acellular porcine corneal scaffolds, and the reconstructed corneal grafts achieved satisfactory outcomes after rabbit xenotransplantation. Furthermore, the corneal endothelium resided in the space between the corneal stroma and lens and developed subsequent to lens formation. This anatomical and developmental feature indicated that stromal keratocytes and lens epithelial cells were capable of regulating the generation of CECs [85, 89]. Table 1 summarizes some recent studies of the differentiation of stem cells into CECs with a coculture method using the Transwell system.

**Table 1:** The differentiation of stem cells into CECs with coculture method using the Transwell system.

Cell source	Method	Culture media	Duration (days)	In vitro	In vivo	Author/year
Human fetal bone marrow‐derived endothelial progenitor cells	Bone marrow‐derived endothelial progenitor cells were cocultured with CECs within a transwell system in the presence of CEC‐derived conditioned medium.	Coculture with CECs was performed in a transwell system. The culture solution was prepared by mixing 5% FBS‐containing Dulbecco’s Modified Eagle Medium (DMEM) and CEC conditioned medium at a 1:1 ratio.	10 (in vitro); 28 (in vivo)	IF: AQP1 IHC: NSE; RT‐PCR: AQP1	Using a decellularized porcine corneal matrix as a scaffold, the induced cells were grafted onto feline corneas that had been denuded of Descemet’s membrane and the endothelium.	Shao et al., 2011 [76]
Human adipose‐derived stem cells (ADSCs)	Coculture human ADSCs with CECs	DMEM/F12, 10% FBS, 0.1 mM nonessential amino acid, 1 mM sodium pyruvate, 2 mM L‐glutamine, 100 µM β‐mercaptoethanol, and 5 ng/mL bFGF.	38	IF: Nanog, vimentin, CD31, AQP‐1, and ZO‐1; RT‐PCR: Nanog	N/A	Dai et al., 2014 [90]
Human ESCs	CECs were derived from human ESCs with CECs differentiation medium.	SV‐40‐transformed human lens epithelial cell‐conditioned medium (LECCM): DMEM/F12, 20% FBS; corneal fibroblast cell (CFC) medium: DMEM/F12, B27, 20 ng/mL EGF, and 40 ng/mL bFGF; CECs medium: LECCM was mixed with CFC medium at a ratio of 3:1	14 (in vitro); 56 (in vivo)	IF: N‐cadherin, ZO‐1, and Na^+^/K^+^‐ATPase FACS: p63	Coculture of corneal endothelial cells and limbal epithelial‐like cells on decellularized porcine corneal matrix; increased corneal transparency at 8 weeks post‐transplantation.	Zhang et al., 2017 [91]
Human skin‐derived precursors	Human SKPs were cocultured with B4G12 cells under serum‐free conditions.	Human endothelial SFM, 10 ng/mL bFGF	8 (in vitro); 3, 7, 30, and 90 (in vivo)	IF: ZO‐1, Na^+^/K^+^‐ATPase WB: ZO‐1, Na^+^/K^+^‐ATPase; RT‐PCR: ZO‐1, Na^+^/K^+^‐ATPase N‐cadherin, carbonic anhydrase type 2 (Car2), COL4A2, COL8A2, Foxc1, and Pitx2	Rabbit and monkey：corneal endothelial cell injection transplantation yields good therapeutic effects.	Shen et al., 2017 [48]
Human skin‐derived precursors	Human SKPs were cocultured with B4G12 cells under serum‐free conditions.	Human endothelial SFM, 10 ng/mL bFGF	10 (in vitro); 21 (in vivo)	IF: Na^+^/K^+^‐ATPase, ZO‐1, and Pitx2 WB: Na^+^/K^+^‐ATPase, Pitx2, Foxc1, AKT, P‐AKT, ERK, P‐ERK, glycogen synthase kinase 3β (GSK‐3β), β‐catenin, active β‐catenin, and p‐GSK‐3β; RT‐PCR: Pax6, Car2, SLC4A4, CDH2, COL4A2, and COL8A2	CEC‐like cells grew well on the APCM scaffold, and the TEC graft performed well after transplantation to the rabbits.	Shen et al., 2024 [88]

### 3.2. Indirect Contact Coculture Using Conditioned Medium Method

In the indirect contact coculture system, the conditioned medium is also frequently used. It refers to cell culture supernatant rich in secretory components secreted by cultured cells [92, 93]. The preparation process involves culturing one cell population first; the harvested supernatant contains diverse bioactive substances including proteins, cytokines, growth factors, chemokines, lipids, and functional mRNA transcripts [94, 95]. Some studies have reported that conditioned medium derived from stem cells can promote the proliferation and function of CECs. When CECs were grown in conditioned medium obtained from ESCs or iPSCs, the proliferative ability of the cells was moderately enhanced, and the number of apoptotic cells also decreased [96, 97]. Likewise, conditioned medium harvested from bone marrow, adipose tissue, and umbilical cord MSCs stimulates CEC proliferation and sustains the differentiated endothelial phenotypes required for normal corneal endothelial function [98–100]. On the contrary, CECs, corneal stromal cells, and lens epithelial cells cultured in vitro secrete multiple inducing factors into culture supernatant. Such collected medium can serve as an induction medium to drive stem cell differentiation toward corneal endothelial lineages [101, 102].

Conditioned medium can be used to induce ESCs or iPSCs to differentiate into CECs, with two‐step induction strategies widely adopted in relevant differentiation protocols. Zhang et al. [102] induced human ESCs to differentiate into POMPs using the Transwell coculture system and then derived CECs from POMPs with LECCM. After transplantation of the CEC‐like cells into rabbit models suffering from corneal endothelial dysfunction, gradual recovery of corneal transparency was observed. LECCM was derived by collecting the medium from SV‐40‐transformed human lens epithelial cells, which plays a crucial role in the final step of CECs derivation. Chen et al. [103] induced human ESCs to differentiate into POMPs using human corneal stromal cell‐conditioned medium and then differentiated into CECs using LECCM plus corneal stromal cell‐conditioned medium. The transition from POMPs to CECs is modulated by the TGFβ‐2/Foxc1 signaling cascade, which can be activated by soluble factors contained in the conditioned medium. Another research focused on mouse ESCs and iPSCs: all‐trans retinoic acid was utilized in the first induction step to stimulate neural crest differentiation. Afterwards, a second round of induction was performed with CEC‐derived conditioned medium or LECCM, eventually generating mature CECs. The expression levels of multiple characteristic CECs proteins and transcripts were significantly upregulated after induction, including ZO‐1, Na^+^/K^+^‐ATPase, AQP1, N‐cadherin, VE‐cadherin, type VIII collagen, vimentin, and SLC4A4 [104]. In the research of Song et al. [101], they first induced H9 hESCs to NCCs. Then, NCCs were incubated in conditioned medium derived from bovine CECs to obtain CECs that express specific markers.

Conditioned medium can also be used to induce MSCs or other stem cells to differentiate into CECs. Joyce et al. [105] used three different culture media including a basal medium of culturing MSCs (MSCBM), a basal medium of culturing lens epithelial cells (LECBM), and LECCM to differentiate umbilical cord blood‐derived MSCs (UCB MSCs) into CECs. These cells expressed ZO‐1 and N‐cadherin when cultured in LECBM or LECCM. In addition, UCB MSCs exhibited the capacity to migrate at sites of corneal endothelial injury, and LECCM provided optimal conditions for this phenotypic transformation. Another study demonstrated that the combination of human CECs‐derived conditioned medium and ROCK inhibitor Y‐27632 could differentiate umbilical cord blood derived from endothelial progenitor cells (UCB EPCs) into CECs. In vivo results demonstrated that combined intracameral injection of differentiated UCB EPCs and Y‐27632 exerted better effects in repairing corneal endothelial injury than separate application of the two agents [106]. In addition, Bosch et al. [107] induced dental pulp stem cells into CECs through suspension culture followed by secondary induction using corneal endothelial conditioned medium, and the resulting polygonal endothelial‐like cells robustly expressed characteristic markers. Table 2 summarizes some recent studies on the differentiation of stem cells into CECs using the conditioned medium method.

**Table 2:** The differentiation of stem cells into CECs using conditioned medium method.

Cell source	Method	Culture media	Duration (days)	In vitro	In vivo	Author/year
Rat NCCs	CECs‐derived conditioned medium	Conditioned medium: medium from cultured rat CECs was mixed with DMEM/F12 at a ratio of 3:1 with 10% FBS.	7, 14 and 20 (in vitro); 7, 14, 21, 28, and 60 (in vivo)	IF: N‐cadherin, ZO‐1, and Na^+^/K^+^‐ATPase; RT‐PCR: Foxc1 and Pitx2 FCM: N‐cadherin	Experimental group: better transparency, thinner cornea, CEC monolayer on Descemet’s membrane vs. control	Ju et al., 2012 [108]
Human UCB MSCs	LECBM LECCM	LECCM was diluted 1:1 in LECBM LECBM: the American Type Culture Collection (ATCC)‐formulated Eagle’s Minimal Essential Medium, 20% FBS, and antibiotic/antimycotic solution diluted 1:100	21 (in vitro); 14 (in vivo)	IF: ZO‐1 and N‐cadherin; RT‐PCR: NCAM1, TFAP2β, CDK15, MEIS1, COL8A1, THY1(CD90), CDH2, and TJP1	LECCM promotes UCB MSC homing and CEC differentiation in injured corneal endothelium ex vivo	Joyce et al., 2012 [105]
Human ESCs and iPSCs	CECs‐derived conditioned medium	Conditioned medium: medium from cultured rat CECs was mixed with DMEM/F12 at a ratio of 3:1 with 10% FBS.	19	IF: ZO‐1; RT‐PCR: COL4A1 and COL8A1	N/A	Fukuta et al., 2014 [109]
Human ESCs	First, human ESCs were induced to differentiate into POMPs with the transwell coculture system. Then, CECs were derived from POMPs with LECCM.	LECCM: DMEM/F12, 20% FBS; FM: DMEM/F12, 10% FBS, B27, 20 ng/mL EGF, 40 ng/mL bFGF; CECs differentiation medium: LECCM was mixed with FM at a ratio of 3:1	28 (in vivo); 14 (in vitro)	IF: Foxc1, Pitx2, ZO‐1 Na^+^/K^+^‐ATPase, N‐cadherin, and vimentin; RT‐PCR: Na^+^/K^+^‐ATPase a1 and β1 subunit WB: Na^+^/K^+^‐ATPase α1 and β1 subunits FACS: vimentin and N‐cadherin	CEC sheets on decellularized porcine posterior corneal lamellae recover corneal transparency in rabbit models	Zhang et al., 2014 [102]
Mouse ESCs line R1 and iPSCs derived from mouse neural progenitor cells	All‐trans retinoic acid induces NCC differentiation from mouse ESCs/iPSCs; subsequent LECCM induction yields CECs	Conditioned medium from CECs or LEC with DM; CECs defined medium: DMEM/F12, 20 ng/mL bFGF, 1% N2 neural supplement, and 2% B27 supplement	17	IF: ZO‐1 Na^+^/K^+^‐ATPase α1, AQP1, VE‐cadherin, N‐cadherin, and vimentin; RT‐PCR: AQP1, Na^+^/K^+^‐ATPase, ZO‐1 N‐cadherin, VE‐cadherin, COL8A1, vimentin, and SLC4A4	N/A	Chen et al., 2015 [104]
H9 human ESCs	Revised two‐step protocol differentiates H9 ESCs into NCCs, which mature into CECs in bovine CEC‐conditioned medium	Conditioned medium from bovine CECs: DMEM/F12 and M199 (1:1), 5% FBS, nonessential amino acids, 2 mM Gluta MAX, 2 ng/mL human recombinant insulin, 20 mg/mL L‐ascorbic acid, 10 ng/mL bFGF	40	IF: Na^+^/K^+^‐ATPase, ZO‐1, Vimentin, AQP1, S100A4, Wnt5a; RT‐PCR: AQP‐1 and COL8A1	N/A	Song et al., 2016 [101]
H9 and H1 human ESCs	Two‐phase induction with corneal stromal and LECCM mixed conditioned media	Conditioned medium from SV‐40 transformed HLEC: DMEM/F12, 10% FBS; CM from CECs (B4G12): human‐endothelial SFM; LECCM was mixed with HCECCM at the ratios of 3:1, 2:1, and 1:1.	23	IF: Foxc1, Pitx2, ZO‐1 Na^+^/K^+^‐ATPase, N‐cadherin, and vimentin WB: Na^+^/K^+^‐ATPase; RT‐PCR: Na^+^/K^+^‐ATPase	N/A	Chen et al., 2018 [103]
Human UCB EPCs	Human CECs‐derived conditioned medium and Y‐27632	CON group: Endothelial cell growth medium‐2; Conditioned medium group): human EGF, human bFGF, VEGF, R3‐insulin‐like growth factor (IGF)−1, hydrocortisone, ascorbic acid, GA‐1000, and 5% FBS; CECs‐derived conditioned medium /Y‐27632 group: CECs‐derived conditioned medium enriched with 10 mM Y‐27632	7–14 (in vitro); 28–42 (in vivo)	IF: ZO‐1 N‐cadherin, Na^+^/K^+^‐ATPase α1 Na^+^/K^+^‐ATPase β1, and Pax6; RT‐PCR: COL4A2 and COL8A2	Rabbit: Intracameral coinjection of UCB EPCs and Y‐27632 promotes faster corneal transparency recovery, more pronounced edema relief and endothelial restoration when compared with single UCB EPC or Y‐27632 injections.	Zhang et al., 2021 [106]
Dental pulp stem cells isolated from human third molars of healthy patients	Suspension‐cultured NCCs differentiate into CECs in corneal endothelial‐conditioned medium	Conditioned medium from human CECs: DMEM high glucose, 1% GlutaMAX, 5% FBS, 2 ng/mL bFGF, 0.1 mM 2‐mercaptoethanol, 10 ng/mL heregulin β, 200 ng/mL IGF‐I, and 10 ng/mL activin A	40	IF: ZO‐1, ATP1A1, COL4A2, and COL8A2; RT‐PCR: ZO‐1, ATP1A1, COL4A2, and COL8A2	N/A	Bosch et al., 2021 [107]

## 4. Induction of Stem Cells Into CECs With Biochemical Induction Methods

Chemically defined induction medium possesses well‐defined component ratios, and its constituents may be modified to accommodate particular cell types. Various growth factors and bioactive substances, including recombinant proteins and small‐molecule compounds, can be incorporated into defined induction media. The chemically defined protocol could induce the proliferation and differentiation of stem cells [110], which would reduce the cost of experiments, be time‐effective, and be repeatable to obtain abundant CECs for clinical applications [85].

The neural crest is an embryonic migratory cell population with the differentiation potential for peripheral neurons, glia, melanocytes, CECs, and more [109, 111]. Previous literatures have demonstrated that NCCs commence migration after undergoing epithelial‐mesenchymal transition, and TGF‐β signaling participates in modulating this cellular transformation process [112]. Two signaling molecules exert ordered regulatory effects on Pitx2: retinoic acid is responsible for inducing gene expression, and subsequent canonical Wnt signaling retains the expression level [113]. The epithelial‐mesenchymal and mesenchymal‐endothelial transition by TGF‐β, bone morphogenetic protein (BMP), and Wnt signaling may have important roles in CECs development [85]. The researchers’ approach was generally to first produce NCCs from ESCs or iPSCs by using dual Smad inhibitors, Noggin (a BMP antagonist) and SB431542 (a TGF‐β inhibitor) [114], and second convert NCCs into CECs [74]. Menendez et al. [115] described that suppressing overall Smad pathway activity while activating the canonical Wnt pathway enables efficient differentiation of NCCs from ESCs or iPSCs. The developmental mechanism of CECs remains poorly understood. McCabe et al. [74] employed a defined medium with B27, platelet‐derived growth factor‐BB (PDGF‐BB), Dickkopf‐related protein 2 (DKK‐2), and bFGF to differentiate NCCs into CECs. Multiple lines of evidence verify that these induced cells share high similarity with adult CECs, providing a cell model for further research on the relevant developmental mechanisms.

Some studies aim to develop strategies for deriving CECs from iPSCs. For example, Wagoner et al. [116] established human iPSCs from adult ocular‐normal fibroblasts via Oct4, Sox2, Klf4, and c‐Myc reprogramming. Subsequent dual inhibition of GSK‐3 and TGF‐β/Smad signaling using CHIR99021 and SB431542 efficiently directed iPSCs toward the NCCs lineage. Further supplementation with B27, PDGF‐BB, DKK‐2, and bFGF drove NCCs differentiation into CECs. The induced cells expressed CEC‐specific genes (ATP1A1, COL8A1/A2, AQP1, and CDH2) from day 20 to day 84 of differentiation. Moreover, early medium replacement on days 3, 5, or 8 significantly shortened the overall CEC differentiation duration. Ali et al. [46, 117] developed CECs from PBMC‐derived iPSCs via a 20‐day protocol modified from the system established by McCabe et al. [74]. Compared with undifferentiated iPSCs, these induced cells exhibited markedly elevated expression of CEC‐specific markers, with barely detectable pluripotency gene expression. Additionally, Proteomic and transcriptomic analyses collectively verified the high similarity of stem cell‐derived CECs to native CECs. The proteome of iPSC‐derived CECs shared 90.82% homology with primary CECs [46], while hESCs‐ and iPSCs‐derived CECs possessed over 96% identical genes, and more than 95% of these genes showed equivalent expression levels [118]. Transplantation of cryopreserved human ESC‐derived CECs into the anterior chamber restored corneal transparency and eliminated edema in rabbit, monkey, and canine corneal endothelial dysfunction models [117, 119]. Chen et al. [120] screened 30 compounds targeting corneal development and stem cell differentiation pathways to induce human iPSC differentiation into CECs and found that the combination of small molecules A769662 and AT13148 effectively produces functional CECs. A769662 facilitates cell migration and differentiation through AMPK activation, while AT13148 exerts identical effects by modulating the PI3K/Akt pathway and Foxc1 expression. Together, these two compounds promote CECs maturation and functionality by regulating core transcription factors Pitx2 and Foxc1. The differentiated cells exhibited high levels of CEC‐specific markers, with morphology and physiological functions comparable to primary CECs. Building on these studies, Kethiri et al. [121] established an optimized unified protocol to generate and purify iPSC‐derived CECs and verified that serum starvation treatment greatly enriches homogeneous iPSC‐CEC populations.

DKK2 encodes an inhibitory protein that antagonizes the Wnt/β‐catenin signaling cascade [122]. During ocular development within NCCs, DKK2 acts as an indispensable downstream target of Pitx2, a homeodomain transcription factor [123].The critical regulatory function of Wnt/β‐catenin signaling in the differentiation of NCCs into CECs can be validated by replacing DKK2 with alternative Wnt pathway antagonists. Jia et al. [124] established a two‐stage induction protocol to generate CECs from human iPSCs. In the first stage, two small molecules, SB431542 and CHIR99021, were supplemented to modulate TGF‐β and Wnt pathways, thereby triggering the differentiation of human iPSCs into NCCs. In the subsequent step, B27, PDGF‐BB, and XAV939 were supplemented to basal CEC culture medium to drive NCC maturation into CECs. XAV939 represents a small‐molecule inhibitor of the Wnt pathway, which selectively represses β‐catenin‐dependent transcriptional activity. It stabilizes axin, the rate‐limiting component of the β‐catenin destruction complex, to block Wnt signal transduction [125]. Pan et al. [126] proposed a two‐step lineage reprogramming strategy. With a defined small‐molecule cocktail, mouse embryonic fibroblasts were sequentially converted into chemically induced NCCs and chemically induced CECs. The team adopted a serum‐free chemical medium containing six small molecules (SB431542, CHIR99021, Forskolin, valproic acid, EPZ004777, and 5‐azacytidine) to reprogram fibroblasts into NCCs. The resultant NCCs were further cultured in medium supplemented with SB431542 and CKI‐7 for CECs differentiation. As a casein kinase I inhibitor, CKI‐7 serves as an effective replacement for DKK2 to suppress Wnt signaling [127]. Transplantation of these induced cells into a well‐characterized rabbit model of bullous keratopathy reversed corneal opacification [126].

Retinoic acid, the bioactive metabolite of vitamin A, acts as a key morphogen for embryonic development. Abnormal retinoic acid levels during gestation lead to multiple ocular developmental defects [128, 129]. Cornea‐derived precursors (COPs) isolated from adult corneal stroma are multipotent stem cells originating from the neural crest [130]. Hatou et al. [50] generated functional tissue‐engineered corneal endothelium from mouse and human COPs. The culture medium contained retinoic acid and a GSK‐3β inhibitor to activate Wnt/β‐catenin signaling. These two additives jointly upregulated Pitx2, a transcription factor responsible for anterior eye segment development. GSK‐3β inhibition also boosted ATP1A1 expression and activity. After transplanting engineered endothelial tissues into rabbit corneas, normal corneal transparency and thickness were well maintained. Grönroos et al. [70] adopted a triple small‐molecule induction system (SB431542, CHIR99021, and retinoic acid) to drive human ESCs and iPSCs toward CECs via neural crest and POM intermediates. Cell identity was validated by typical morphology and positive expression of CECs biomarkers. Retinoic acid further induced dome‐like structures in cultured cells, a marker of fluid transport function. Wnt/β‐catenin signaling determines neural crest axial identity in a dose‐dependent manner. Retinoic acid and bFGF collaboratively control Hox gene expression in posterior NCCs [131]. Nevertheless, bFGF exerts adverse impacts on CECs maturation in vitro by triggering EMT [132–134]. Yu et al. [134] verified that bFGF treatment blocked NCC differentiation into mature CECs with ZO‐1 and Na^+^/K^+^‐ATPase expression. Combined inhibition of TGF‐β, Wnt, and FGFR pathways with SB431542, DKK2, and SU‐5402, respectively, induced mature CECs from NCCs within 12 days. The differentiated cells highly expressed ZO‐1, Na^+^/K^+^‐ATPase, N‐cadherin, and CD166. Only cells passing full quality control could be used for subsequent in vivo research. Zhao and Afshari [135] optimized the induction scheme for ocular NCSCs. They removed bFGF from differentiation medium, inhibited TGF‐β signaling with SB431542, and added H‐1152 to suppress ROCK activity and expand target cells. The induced CECs expressed standard endothelial biomarkers; H‐1152 showed stronger ROCK inhibitory capacity than Y27632. The findings of So et al. [136] further optimized iPSC‐to‐CECs differentiation. The combination of vitronectin coating and fasudil (a synthetic ROCK inhibitor superior to Y27632 in operability) achieved differentiation.

Most iPSC‐based ocular differentiation protocols yield only a single cell type and fail to recapitulate eye developmental complexity. Li et al. [137] constructed an efficient 2.5D culture system to derive multizone ocular cells (MZOCs) from iPSCs. The surface ectoderm fraction of MZOCs differentiates into corneal epithelial cells, whereas its neural crest fraction generates CECs. Conventional induction systems for generating CECs from ESCs and iPSCs adopt multistage procedures that mimic ocular developmental trajectories via NCCs intermediates, hindering large‐scale cell fabrication. Hatou et al. [49] constructed a rapid culture workflow enabling direct CECs derivation from iPSCs without the standard NCCs differentiation step. The xeno‐ and human component‐free chemically defined medium containing tailored cytokines modulates the transcriptional abundance of C‐Jun, C‐Fos, NR3C2, and Sox9 to activate corneal endothelial developmental programs. Cryopreserved iPSC‐originated CECs were transported, resuscitated, and grafted into primate corneal edema models. Relative to control groups, eyes receiving CECs grafts exhibited prominent alleviation of corneal swelling.

Great progress has been made in generating CECs from ESCs and iPSCs, yet such cell lines carry drawbacks including oncogene utilization and difficult construction. Alternative research explores small‐molecule‐based induction of MSCs into CECs. Marta et al. [138] optimized four existing induction protocols to derive CECs from human adipose mesenchymal stem cells (ADSCs). The scheme reported by Ali et al. [46] delivered superior performance in differentiation efficiency, culture cycle, and experimental cost, while Wagoner et al.’s protocol [116] yielded cells with the highest expression of CEC biomarkers. Inagaki et al. [139] adopted medium supplemented with retinoic acid and the GSK‐3β inhibitor BIO to induce human skin‐derived precursors (SKPs) into polygonal CECs. After transplantation into rabbit bullous keratopathy models, these cells preserved normal corneal thickness and transparency. Yamashita et al. [140] treated human umbilical cord mesenchymal stem cells (UC‐MSCs) with BIO‐containing medium to generate polygonal CEC‐like cells. Tissue‐engineered endothelium constructed from these cells maintained corneal thickness and transparency in rabbit bullous keratopathy models. Ye et al. [47] induced human UC‐MSCs to differentiate into CECs using medium with BIO plus two ROCK inhibitors (Y‐27632 and H‐1152). In rabbit corneal endothelial injury models, injection of MSC‐derived CECs relieved corneal opacity and neovascularization compared with blank controls and pure MSC injection groups. Detection of human mitochondrial DNA in the central cornea confirmed stable cell engraftment. Shin et al. [141] investigated the effects of Y‐27632 on human corneal endothelial progenitor cells (CEPs). Y‐27632 facilitated isolation and expansion of CEPs and elevated the proliferation, viability, and migration capacity of differentiated CEPs.

In brief, several small‐molecule compounds or recombinant proteins have been utilized to generate stem‐cell‐derived CECs via approaches with greater chemical definition compared with earlier protocols. Stem cell‐derived CECs were phenotypically verified by morphological features, canonical corneal endothelial markers, and transcriptomic and proteomic microarray profiling. The characteristics of the chemical induction procedure trace complicated signaling pathway processes in corneal endothelial cell development. Table 3 summarizes some recent studies on the differentiation of stem cells into CECs by biochemical induction methods.

**Table 3:** The differentiation of stem cells into CECs by biochemical induction methods.

Cell source	Method	Culture media	Duration (days)	In vitro	In vivo	Author/year
Mouse and human corneal stroma MSCs (COPs)	Functional corneal endothelial grafts were successfully fabricated from both murine and human COPs.	Eagle’s Minimum Essential Medium containing FBS, 1 mM all‐trans retinoic acid, 1 mM GSK‐3β inhibitor, 5 ng/mL TGF‐β2, 10 mM Y‐27632	7 (in vitro); 8 (in vivo)	IF: ZO‐1 and Na^+^/K^+^‐ATPase; RT‐PCR: ATP1A1, SLC4A4, COL4A2, COL8A2, and CDH2 Measurement of Na^+^/K^+^‐ATPase pump function	Rabbit corneas and human corneas; maintained corneal transparency and thickness	Hatou et al., 2013 [50]
Human corneal endothelial progenitor cells	A novel serum‐free culture system	The medium: DMEM/F12 containing 20% knockout Serum Replacement, and 4 ng/mL bFGF	14–28 (in vitro); 28 (in vivo)	IF: Na^+^/K^+^‐ATPase and ZO‐1; RT‐PCR: p75NTR, Sox9, AP‐2β, snail, slug, Pitx2, Foxc2, Nestin, COL8A1, and COL8A2	In vivo grafting of differentiated HCEP sheets to rabbit corneal tissue conferred corneal endothelial biological function.	Hara et al., 2014 [142]
Human ESC lines H1 Oct4 eGFP, H9, Ma09 and NED07	The first step was to produce NCCs from ESCs; The second step was to convert NCCs into CECs with PDGFB, DKK2, and FGF2.	Dual Smad induction media: 500 ng/mL Noggin and 10 mM SB431542 Cornea media: 0.1× B27 supplement, 10 ng/mL recombinant human PDGF‐BB, and 10 ng/mL recombinant mouse DKK‐2	18	IF: ZO‐1 and ATP1A1 WB: COL8A1/A2; RT‐PCR: NGFR, Sox10, Foxc1, COL8A1/A2, and AQP1	N/A	McCabe et al., 2015 [74]
Human UCB EPCs	Magnetic‐guided UCB EPC transplantation in rabbit anterior chamber	EGM‐2 culture medium, 5%–10% FBS, hydrocortisone, human FGF‐B, VEGF, R3‐IGF‐1, ascorbic acid, human EGF, and GA‐1000	120 (in vivo)	IF: AQP1	CD34 immunomagnetic nanoparticle‐functionalized UCB EPCs home magnetically and repair rabbit corneal endothelial lesions	Shao et al., 2015 [143]
Human PSCs H9 (WA9, WiCell) and iPSC derived from BJ human fibroblasts	TGF‐β and ROCK signaling inhibition drives ocular NCSC differentiation into CECs	Crest induction medium: DMEM/F12, 3 μM CHIR 99021. Cornea media: human endothelial‐SFM, 5% FBS, 1 μM SB431542, and 2.5 μM H‐1125	14	IF: HNK‐1 NGF receptor (p75NTR), β‐catenin, Sox2, ZO‐1 Na^+^/K^+^‐ATPase, and N‐cadherin; RT‐PCR: Pax6, Rax, Lhx2, Six3, Six6, ATP1A1, and NCAD	N/A	Zhao and Afshari 2016 [135]
Mouse SKPs	Skin‐derived precursors were induced to differentiate into functional CECs	Cornea medium: MEM, 5% FBS, 1 mM all‐trans retinoic acid, 0.5 mM BIO, 5 ng/mL TGF‐β2, and 10 µM Y‐27632	7 (in vitro); 8 (in vivo)	RT‐PCR: ATP1A1, CDH2, Pitx2, SLC4A4, COL4A2, and COL8A2 IF: ZO‐1 and Na^+^/K^+^‐ATPase WB: ATP1A1 and Pitx2	CEC‐like cells transplanted into a rabbit model of bullous keratopathy successfully maintained corneal thickness and transparency	Inagaki et al., 2017 [139]
iPSCs from fibroblasts of adults with normal ocular history	NCCs were differentiated from iPSCs; NCCs were differentiated into CECs	NCCs medium: 10 µM SB431542, and 3 µM CHIR99021. Cornea medium: DMEM/F12, and 10 ng/mL recombinant human DKK‐2	~25–96	IF: p75/NGFR, Sox10, ZO‐1 N‐cadherin, AQP1, and ATP1A1; RT‐PCR: PAX3, Sox9, TFAP2A, Sox10, p75NTR, ATP1A1, COL8A1, COL8A2, AQP1, and N‐cadherin	N/A	Wagoner et al., 2018 [116]
Human iPSCs from PBMCs	Noggin/SB431542 induce iPSCs into NCCs; PDGFB/DKK2/FGF2 further differentiate NCCs into CECs	NCCs medium: 500 ng/mL recombinant human Noggin and 10 μM SB431542. Cornea medium: 10 ng/mL recombinant human PDGF‐BB and 10 ng/mL recombinant human DKK‐2	20	RT‐PCR: NGFR, Sox10, AQP1, ATP1A1/A3, COL4A1/A3, COL8A1/A2, Foxc1, and SLC16A3 WB: ZO‐1	N/A	Ali et al., 2018 [46]
H9 human ESCs and human PBMC‐originated iPSCs	The first step was to produce NCCs from ESCs or iPSCs. The second step was to convert NCCs into CECs.	NCCs medium: 500 ng/mL recombinant human Noggin and 10 μM SB431542. Cornea medium: 10 ng/mL recombinant human PDGF‐BB and 10 ng/mL recombinant human DKK‐2	20	IF: ZO‐1 and N‐cadherin	N/A	Ali et al., 2018 [118]
Human UC‐MSCs	UC‐MSCs differentiatedpolygonal CECs with medium containing GSK‐3β inhibitor	Cornea medium: MEM, 0.5 mM BIO, 10 mM Y‐27632, 4 nM triiodothyronine, 0.5 mg/mL hydrocortisone, 50 mg/mL ascorbic acid	9–11 (in vitro); 8 (in vivo)	IF: ZO‐1, ATP1A1; RT‐PCR: ATP1A1, CDH2, Pitx2, COL4A2, SLC4A4, Car2, and COL8A2 WB: Pitx2 and ATP1A1	Tissue‐engineered corneal endothelium restores corneal morphology in bullous keratopathy rabbits	Yamashita et al., 2018 [140]
Human iPSCs	CECs were derived from autologous iPSCs via NCCs	NCCs medium: 500 ng/mL Noggin and 10 mM SB431542. Cornea medium: HES medium, 10 ng/mL human recombinant PDGF‐BB, and 10 ng/mL recombinant mouse DKK‐2	10 (in vitro); 21 (in vivo)	IF: ZO‐1 N‐cadherin, and CD166; RT‐PCR: SLC4A11	N/A	Brejchova et al., 2019 [144]
Human iPSC line DYR0100	The first step differentiated multizone ocular cells from human iPSCs; The second step differentiated neural crest zone into CECs	Cornea medium: 1:1 mixture of defined keratinocyte serum‐free medium and DMEM/F12, 5% knockout serum replacement, 2 ng/μL human EGF, 10 μM Y‐27632	35	IF: Na^+^/K^+^‐ATPase and ZO‐1 FACS: Na^+^/K^+^‐ATPase; RT‐PCR: Na^+^/K^+^‐ATPase, ZO‐1, AQP1, SLC4A4, and SLC16A4	N/A	Li et al., 2019 [137]
H9 human ESCs	The first step was to produce NCCs from ESCs; The second step was to convert NCCs into CECs	NCCs medium: 500 ng/mL recombinant human Noggin and 10 μM SB431542. Cornea medium: 10 ng/mL recombinant human PDGF‐BB and 10 ng/mL recombinant human DKK‐2	20 (in vitro); 21 (in vivo)	IF: Na^+^/K^+^‐ATPase and ZO‐1; RT‐PCR: AQP1, ATP1A1, ZO‐1	Anterior chamber injection of cryopreserved human ESC‐derived CECs restores healthy corneas in rabbit and monkey endothelial dysfunction models	Ali et al., 2021 [117]
Human iPSCs	The first step was to produce NCCs from iPSCs; The second step was to convert NCCs into CECs	NCCs medium: 2 μM SB431542, and 2 µM DMH1. Cornea medium: 10 μM A769662, 10 μM AT13148, 20 ng/mL EGF, and 2 µM CHIR99021	22	IF: Na^+^/K^+^‐ATPase, AQP1, ZO‐1, and vimentin; RT‐PCR: Sox9, Sox10, NTRK3, NGFR, AQP1, ZO‐1, and COL8A1 WB: PKA/AKT signaling pathway, Foxo1, Pax6, Pitx2, and Foxc1	N/A	Chen et al., 2021 [120]
Research grade iPSC clones Ff‐MH09s01, Ff‐I01s04, and clinical grade QHJI01s04	Differentiate iPSCs directly into CECs	Cornea medium: DMEM/F12, 10 ng/mL IGF1, 10 ng/mL leukemia inhibitory factor, 10 ng/mL Interleukin 6, 5 ng/mL IL11, and 10 ng/mL tumor necrosis factor‐α	35 (in vitro); 2 (in vivo)	IF: ATP1A1, ZO‐1 N‐cadherin, and Pitx2	Frozen‐thawed iPSC‐CECs relieve corneal edema in primate models	Hatou et al., 2021 [49]
Human ESC line Regea08/017 and human iPSC line WT001.TAU.bB2	Differentiate human iPSCs through NCCs and periocular mesenchyme to generate CECs	Cornea medium: DMEM, 15% knockout serum replacement, 500 nM BMP pathway inhibitor LDN193189, 10 µM SB431542, 4 µM CHIR99021, and 10 µM retinoic acid	7	IF: Sox10, AP2α, Pax6, ZO‐1 Na^+^/K^+^‐ATPase, CD166, and Foxc1; RT‐PCR: Pitx2, AQP1, ATP1A1, CDH2, SLC4A4, and Foxc1	N/A	Gronroos et al., 2021 [70]
Human iPSCs	The first step was to produce NCCs from iPSCs; The second step was to convert NCCs into CECs	NCCs medium: 20 ng/mL bFGF and 20 ng/mL EGF. Cornea medium: 8 ng/mL bFGF, 10 ng/mL recombinant human PDGF‐BB, 10 ng/mL recombinant human DKK2, 1 mM SB431542, and 2.5 mM Y27632	26 (in vitro); 28 (in vivo)	IF: NSE, p75, vimentin, Na^+^/K^+^‐ATPase, and ZO‐1; RT‐PCR: p75, vimentin, ATP1A1, and ZO‐1	The restoration of corneal clarity was successfully facilitated by differentiated CECs.	Sun et al., 2021 [145]
Mouse embryonic fibroblasts	The first step was to produce NCCs from ESCs; The second step was to convert NCCs into CECs	Cornea medium: DMEM/F12, 10% knockout serum replacement, 5 μM SB431542, and 5 μM CKI‐7	30–35 (in vitro); 7–42 (in vivo)	IF: P75, HNK1, AP2α, Nestin, Na^+^/K^+^‐ATPase, AQP1, ZO‐1, and N‐cadherin; RT‐PCR: Na^+^/K^+^‐ATPase, AQP1, and N‐cadherin	Rabbit model with bullous keratopathy; reversed corneal opacity	Pan et al., 2021 [126]
Human ADSCs	Human ADSCs were induced to differentiate into NCCs and then into CECs	NCCs medium: DMEM, 1% FBS, 100 ng/mL FGF2, 10 μM forskolin The Ali et al. [46] protocol The Wagoner et al. [116] protocol	30–40	IF: S100, Na^+^/K^+^‐ATPase; RT‐PCR: S100, Na^+^/K^+^‐ATPase, ZO‐1, and AQP1	N/A	Marta et al., 2021 [138]
Human ES cell line H1, human iPS cell line DYR0100 and U2	A two‐stage protocol to differentiate NCCs and CECs from human ESCs and iPSCs	NCCs medium: DMEM/F12, 20% knockout serum replacement, 4 ng/mL bFGF, and 1 μM retinoic acid. Cornea medium: DMEM/F12, 0.2% BSA, 8 ng/mL bFGF, 10 μg/mL PDGF‐BB, and 10 μg/mL DKK‐2	19 (in vitro); 14–56 (in vivo)	IF: P75, HNK‐1, AP‐2α, AP‐2β, ZO‐1, ATP1A1, and AQP1 FACS: P75, HNK‐1, ATP1A1, and SLC4A11; RT‐PCR: P75, AP‐2α, ZO‐1, and ATP1A1	Nicotinamidesupplemented corneal endothelial progenitors restore corneal clarity/thickness in rabbit and NHP models	Li et al., 2022 [146]
Human iPSCs	The first step was to produce NCCs from iPSCs; The second step was to convert NCCs into CECs	NCCs medium: 1:1 mixture of Iscove’s modified Dulbecco’s medium and F12, 5 µg/mL bFGF, 5 mg/mL BSA, 10 µM SB431542, and 0.5 µM CHIR99021. Cornea medium: 10 ng/mL PDGF‐BB and 0.5 μM XAV939	24	IF: Sox10, β‐catenin, and ZO‐1; RT‐PCR: Sox9, Sox10, NGFR, HNK‐1, β‐catenin, COL4A1, COL8A1, COL8A2, and ZO‐1	N/A	Jia et al., 2022 [124]
Human iPSC	iPSCs were converted into CECs.	Day 1: 100 nM BGJ398, 500 nM LDN19318 Day 2: E6 basal medium, 1400 nM CHIR99021, 100 ng/mL bFGF, and 250 nM SAG Day 3: E6 medium supplemented with 100 nM BGJ398, 20 ng/mL BMP4 Day 4: E6 medium, 10 ng/mL bFGF, Cornea medium: human endothelial‐SFM, 1 μM SB431542, 2.5 μM ROCK inhibitor H‐1152, and 10 μM Y27632	17 (in vitro); 21 (in vivo)	IF: ATP1A1, ZO‐1, and SLC4A11; RT‐PCR: Oct4, Sox2, Nanog, p75NTR, PAX3, ATP1A1, COL8A1, and AQP1 WB: ZO‐1 NCAM, SLC4A11, N‐cadherin, and ATP1A1	Injection of iPSC‐derived CEC‐like cells into the anterior chamber of a corneal endothelial dysfunction rabbit model restored corneal clarity.	So et al., 2022 [136]
Human UC‐MSCs	Differentiate human UC‐MSCs into CECs directly	Cornea medium: Human endothelial‐SFM, 0.5 mM BIO, 10 µM Y‐27632, 1 μM SB431542, 2.5 μM H‐1152	16 (in vitro); 21 (in vivo)	IF: ATP1A1, ZO‐1 NCAM, and CD166; RT‐PCR: ATP1A1, COL8A1, AQP1, and COL8A2 FACS: CD166	MSC‐derived CECs improved the corneal opacity and neovasculariz ation of rabbit model of corneal endothelial dysfunction	Ye et al., 2022 [47]
The human ESCs lines CB0017, CB0018, and CB0019	First, human ESCs were induced to differentiate into NCCs. The second step was to convert NCCs into CECs	Peripheral neuron differentiation medium: N2 Supplement, B27 Supplement XenoFree, 200 µM ascorbic acid, and 2 µM dibutyryl‐cAMP Cornea medium: knockout DMEM/F12, knockout SR XenoFree, GlutaMAX, ascorbic acid, 10 µM Y27632, and 10 µM SB431542	24 (in vitro); 28 (in vivo)	IF: ZO‐1 Na^+^/K^+^‐ATPase and AQP1; RT‐PCR: B3GAT1, NES, NGFR, Sox10, TFAP2A, TJP1, CDH2, and AQP1 RNA sequencing (RNA‐seq): ATP1A1 and CLCN3Trans endothelial electrical potential difference	No hyperplasia/tumor in immunocompromised mice; viable CECs reverse corneal swelling and restore transparency in rabbits	Yu et al., 2023 [134]
Human CEPs	Response of differentiated CEPs to varying concentrations of Y‐27632	Culture medium: DMEM/F12, knockout serum replacement, 100 μM 2‐mercaptoethanol, 50 U/mL penicillin G, 50 μg/mL streptomycin, 4 ng/mL bFGF, and 10 μM Y‐27632 Differentiation medium: low‐glucose DMEM, FBS, penicillin G, and streptomycin	15	IF: p75NTR, Sox9, ZO‐1, and Na^+^/K^+^‐ATPase WB: ZO‐1 and Na^+^/K^+^‐ATPase	N/A	Shin et al., 2023 [141]
Human ESC line Regea08/017 and human iPSC line WT001.TAU.bB2	Human ESCs and iPSCs were directed toward NCCs and POM lineages	Induction medium: knockout DMEM, 15% knockout serum replacement, 2 mM GlutaMax‐I, nonessential amino acids, 10 µM TGF‐β inhibitor SB431542, 4 µM GSK3 inhibitor/Wnt pathway activator CHIR99021, and 10 µM retinoic acid	7–9	IF: ZO‐1 Na^+^/K^+^‐ATPase and CD166; RT‐PCR: CD166 FACS: CD166	N/A	Gronroos et al., 2024 [147]
H9 human ESCs	The first step was to produce NCCs from ESCs; The second step was to convert NCCs into CECs	Dual Smad inhibitors media: 500 ng/mL human recombinant Noggin and 10 μM SB431542 Cornea medium: B27, 10 ng/mL recombinant human PDGF‐BB, and 10 ng/mL recombinant human DKK‐2	20 (in vitro); 90 (in vivo)	IF: N‐cadherin, Na^+^/K^+^‐ATPase α1, and ZO‐1	Intracameral injection of cryopreserved human ESC‐CECs reconstructs functional corneal endothelium on canine Descemet’s membrane.	Ali et al., 2025 [119]
Human iPSCs	A two‐stage protocol for directing human iPSC differentiation into NCCs and CECs	Dual Smad inhibitor medium: 500 ng/mL recombinant human Noggin and 10 μ M SB431542 Corneal medium: 10 ng/mL recombinant human PDGF‐BB, 10 ng/mL recombinant human DKK‐2, and B27 supplement	7–20 (in vitro); 28 (in vivo)	IF: SOX10, NGFR, ZO‐1, and Na^+^/K^+^‐ATPase WB: NGFR, ZO‐1, and Na^+^/K^+^‐ATPase	Thermosensitive hydrogel combined with iPSC‐CECs repairs rabbit corneal endothelial dysfunction	Chi et al., 2025 [122]
Human ESC line Regea08/017 and human iPSC line WT001.TAU. bB226,28 and AICS‐0016−184 iPSC line	Differentiation and enrichment protocol of human iPSC‐CECs in SB431542, CHIR99021, and retinoic acid‐supplemented serum‐free medium	Differentiation medium: Knockout DMEM basal medium supplemented with knockout serum replacement, 10 μM SB431542, 4 µM CHIR99021, and 1 µM retinoic acid.	13	IF: CD166, Na^+^/K^+^‐ATPase, ZO‐1, AP2α, and N‐cadherin	N/A	Kethiri et al., 2025 [121]
PBMC‐derived human iPSCs	The first step was to produce NCCs from PBMC‐derived human iPSCs. The second step was to convert NCCs into CECs	Dual Smad inhibitor medium: basal medium supplemented with 500 ng/mL recombinant human Noggin and 10 μM SB431542. Cornea medium: medium containing B27 supplement, 10 ng/mL recombinant human PDGF‐BB, and 10 ng/mL recombinant mouse DKK‐2.	9–32 (in vitro); 56 days (short‐term); 210 days (long‐term) (in vivo)	IF: ZO‐1, ATP1A1, COL8A1, AQP1, and CD166	Transplantation of hiPSC‐derived corneal endothelial progenitor‐like cells rescues BAK‐induced corneal endothelial dystrophy in rabbits	Hsueh et al., 2025 [148]
Human iPSCs (QHJI01s04)	Directed differentiation into corneal endothelial substitutes (CLS001)	Differentiation medium: DMEM/F12, KSR, N2, B27, and 8 ng/mL bFGF 10 ng/mL PDGF‐BB, 10 ng/mL DKK‐2, 10 μM Y‐27632 (ROCK inhibitor), NEAA, 2 mM GlutaMAX, and 0.1 mM β‐mercaptoethanol	14 (in vitro); 2 years (in vivo)	IF/FACS: ATP1A1, ZO‐1 N‐cadherin, PITX2, and mineralocorticoid receptor; RT‐PCR/back‐culture test: no residual undifferentiated iPSCs	Animal: Nude rats; tumorigenicity, systemic distribution, residual cells, and organ toxicityHuman: best corrected visual acuity, corneal thickness, IOP, edema, safety, and adverse events	Hirayama et al., 2025 [29]

## 5. Induction of Stem Cells Into CECs With Mechanical Methods

The aforementioned studies indicate that biochemical methods and coculture approaches can induce stem cells to differentiate into CECs. The cellular microenvironment governs cell fate, and biophysical cues constitute a vital component of this microenvironment [149, 150]. Existing studies exploit biophysical cues to probe the intrinsic characteristics and biological behaviors of stem cells, elucidate core mechanobiological mechanisms, and delineate the regulatory pathways governing the lineage‐specific differentiation of stem cells [151, 152]. These biophysical and mechanical factors include cell–cell and cell–extracellular matrix (ECM) interactions, cellular elasticity, contractile forces, shear stress, strain, dynamic tensile forces, and microtopography [153]. The complexity of biological systems within the body has created a need for more advanced *in vitro* technologies. Various bioreactors have been developed that can mimic the mechanical microenvironment of cells [154, 155]. Using these bioreactors, studies from our group and others have demonstrated that mechanical factors can influence the differentiation of stem cells into osteoblasts, adipocytes, endothelial cells, and neurons, among other cell types [156–158]. CECs also exist within a mechanical microenvironment under physiological conditions *in vivo*. *In vitro*, the mechanical factors can influence CECs behavior and function. For example, research by Muhammad et al. [159] confirmed that micro/nanotopographical cues boost cell proliferation and preserve functional marker expression in primary human donor CECs. Mimicking the biophysical and mechanical factors influences stem cell differentiation toward CECs, offering a potential innovative direction for regenerative medicine in CECs.

Some studies have reported the effects of substrate topography and matrix stiffness on the differentiation process of stem cells toward CECs. For example, human MSCs adhered to Descemet membrane‐like microtopography (DLT) acquired polygonal morphology. It can simultaneously upregulate the transcription of characteristic CECs genes (ZO‐1, Na^+^/K^+^‐ATPase, Pitx2, Col‐8) and boost the expression of their associated proteins [160]. Further, this team confirmed this mechanotransductive system by using preinduced Wharton jelly‐derived MSCs. The cells, grown on collagen with DLT, adopted the hexagonal shape typical of CECs and showed significant expression of key genes and proteins. Meanwhile, these cells can establish a potential difference of ~40 mV and sustain transparency of the cornea [161]. Recently, hair follicle stem cells from aged eyelid skin were cultured on a DLT‐imprinted membrane under serum‐free conditions, without additional biochemical factors, and successfully differentiated into CECs, which expressed CECs markers at both the gene and protein levels. Epidermal hair follicle stem cells from eyelid tissue can be harvested via skin biopsy. These cells are applicable for autologous corneal endothelial replacement in patients of all age groups and possess promising clinical application prospects [162]. Researchers found that higher cell density and collagen substrates, rather than smooth silicone, enhanced MSCs differentiation into CECs by increasing the expression of key corneal endothelial genes. This indicates that both substrate stiffness and topography significantly affect the differentiation of MSCs into CECs [160]. The differentiation of stem cells into CECs by mechanical methods in recent years is shown in Table 4.

**Table 4:** The differentiation of stem cells into CECs by mechanical methods.

Cell source	Method	Culture media	Duration (days)	In vitro	In vivo	Author/year
Human foreskin‐derived MSCs	DLT induces MSCs to differentiate into CECs	Culture medium: DMEM, 4.5 g/L glucose, and 10% FBS Dietary medium: DMEM, 4.5 g/L glucose, and 1% FBS	24	IF and RT‐PCR: Na^+^/K^+^‐ATPase, ZO‐1, Pitx2, and Col‐8	N/A	Gutermuth et al., 2019 [160]
Human wharton jelly‐derived MSCs	DLT drives preinduced Wharton’s jelly‐derived MSCs to undergo differentiation toward CECs	Endothelial growth medium 2: DMEM, 20% FBS, and 0.1% CFA Human endothelial serum‐free medium: DMEM, 20 ng/mL bFGF, and 10 ng/mL EGF	16 (in vitro); 5 (in vivo)	IF and RT‐PCR: ZO‐1, Na^+^/K^+^‐ATPase, Pitx2, and Col‐8 Measurement of electrochemical potential difference	Rabbit: cell function assessment via electrophysiology and corneal transparency	Feiertag et al., 2020 [161]
Human eyelid‐derived epidermal hair follicle neural crest stem cells	DLT induces hair follicle stem cells to differentiate into CECs	Endothelial serum‐free medium supplemented with 20 ng/mL bFGF, 20 ng/mL of EGF, and 0.1% ciprofloxacin	24	IF: Pitx2, Na^+^/K^+^‐ATPase, and ZO‐1 RT‐PCR: Nang, Sox‐2, Pitx2, ATP1A1, COL8A1, and ZO‐1	N/A	Olszewski et al., 2022 [162]
Human dental follicle stem cells	Porcine corneal ECM‐cultured dental follicle stem cells differentiate into keratocyte‐like cells and CECs	Minimum essential medium α supplemented with 10% FBS	21	IF: Na^+^/K^+^‐ATPase and N‐cadherin	N/A	Lin et al., 2025 [163]

## 6. In Vivo and Clinical Applications of Stem Cell‐Derived CECs

In vivo experiments are essential before clinical application. Corneal reconstruction utilizing cultured CECs is feasible (Figure 3). At present, Scholars have outlined two core technical routes to implement CEC transplantation. The first is direct cell injection into the anterior chamber, and the second involves culture of CECs on implantable, biocompatible, and biodegradable tissue‐engineered grafts [8]. Reconstructed corneas composed of cultured human CECs and corneal stroma exhibit ~55%–75% of the pump function of normal corneas. Porcine corneal stroma did not trigger hyperacute rejection after transplantation into animal corneas [164]. Human corneal endothelial precursor cells could be successfully transplanted onto the corneal endothelium of bovine eyes employing a nanocomposite gel sheet [165]. In addition, injecting human CECs supplemented with a ROCK inhibitor into the anterior chamber of patients with bullous keratopathy could increase the density of CECs, normalize corneal thickness, resolve corneal endothelial edema, and improve visual acuity [16].

**Figure 3:**
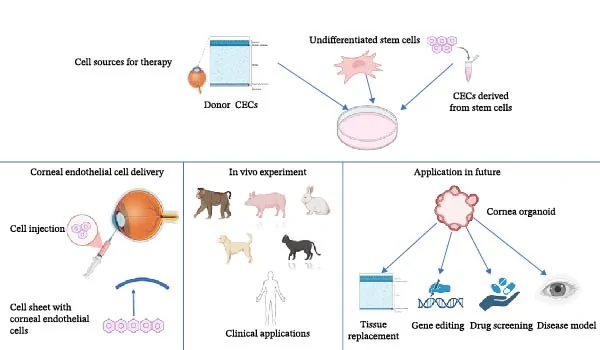
In vivo and clinical applications of stem cell‐derived CECs. Created with BioRender.com.

Some studies suggest that undifferentiated stem cells can promote corneal repair *in vivo* [63]. For example, Fei et al. [166] found that CD44^+^/CD105^+^ MSCs isolated from mouse amniotic fluid, when injected into the corneal endothelium of cryoinjured mice, modulated the corneal microenvironment, promoted endogenous CECs proliferation, and upregulated mRNA expression of CECs markers including ATP1A1 and ZO‐1.

Other researchers revealed that differentiating stem cells into CECs before transplantation might be more conducive to corneal endothelial recovery [63]. These stem cell‐derived CECs are important for the development of treatment technologies and act as the most favorable cell resource for cellular therapeutics of corneal endothelial impairment [167]. A critical indicator of successful differentiation is the efficacy and safety observed in in vivo models [19]. As previously mentioned, stem cell‐derived CECs transplantation has demonstrated therapeutic effects in various animal models, including rabbits [47, 102, 122, 126], monkeys [48, 49, 117], and canines [119] (see Tables 1–4). Hirayama et al. [29] generated CECs substitute using the clinical‐grade human iPSC line CLS001, which were subsequently transplanted into a patient suffering from recurrent bullous keratopathy. One‐year follow‐up data revealed partial alleviation of corneal edema and improvements in corneal transparency and visual acuity, with no adverse clinical reactions observed throughout the period [168, 169]. A long‐term follow‐up study approved by the institutional review board is ongoing for up to 5 years after transplantation. iPSC‐based cell therapies have advanced rapidly in recent years and represent a promising therapeutic strategy. A major safety concern arises from residual undifferentiated cells, which may lead to teratoma formation [8]. iPSCs have been reported to overexpress multiple oncogenes, including RAC3, RAB25, PIM2, MYBL2, and TET1 [170]. Therefore, rigorous tumorigenicity assessment is essential for clinical translation [171, 172].

In addition, iPSC‐derived corneal organoid can recapitulate sophisticated corneal tissue structures and support research focusing on biofabricated transplantable grafts [173]. Foster et al. [174] generated human iPSC‐derived organoids via stepwise differentiation, which contained three major corneal cell types expressing key epithelial, stromal, and endothelial cell markers and deposited stromal ECM collagen fibrils. Corneal organoids constitute a robust three‐dimensional platform for disease modeling and drug screening, with prospective utility in gene editing and tissue‐replacement therapies [175].

## 7. Conclusions and Prospects

In conclusion, this review focuses on the methods for differentiating stem cells into CECs (Figure 4). In vitro engineered CECs models facilitate ophthalmic drug screening and translational research of cell therapeutics for corneal diseases. Owing to the low proliferative potential of primary CECs, endothelial regeneration remains a formidable clinical challenge, while stem cell interventions serve as a promising strategy to rescue damaged corneal endothelial function. These regenerative therapies circumvent core drawbacks of traditional clinical treatments, such as insufficient donors and microbial or immune risks that reduce the long‐term survival of grafted tissues [30].

**Figure 4:**
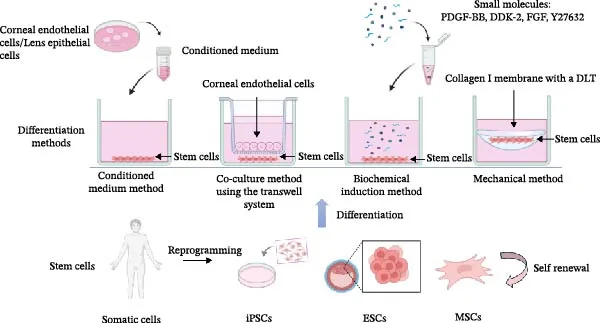
The methods of differentiation from stem cells into CECs. Created with BioRender.com.

*In vitro* differentiation of stem cells into CECs is commonly achieved through chemical induction, physical induction, and coculture with corneal cells, with successful induction validated by morphology, CEC‐specific markers, and lineage‐related gene expression. These three strategies differ considerably in efficiency, purity, timeline, and safety. Coculture systems yield relatively high differentiation efficiency and reliable in vivo function but often rely on feeder cells or conditioned medium, with risks of contamination and inconsistent purity. In recent years, chemical induction has become increasingly mainstream, replacing serum or conditioned medium with recombinant proteins and small molecules [85, 176]. This approach enables scalability, GMP compliance, and low contamination risk, yet suffers from variable efficiency, long induction periods, and potential off‐target effects. Physical induction better mimics the in vivo microenvironment and shows low immunogenicity, making it widely used in MSCs differentiation; however, it is limited by poor standardization and insufficient long‐term functional evidence. Overall, coculture remains effective for producing functional CECs, whereas chemical and physical methods show greater potential for clinical translation owing to superior safety and controllability (Table 5). Combinatorial induction using multiple strategies may represent a future trend.

**Table 5:** The comparison of induction methods for differentiating stem cells into CECs.

Method	Stem cells type	Efficiency	Purity	Duration (days)	Safety	Author/year
Coculture	ESCs	Successful directed differentiation from hESCs to CECs via a two‐step conditioned medium protocol.	90%–95%, hexagonal morphology; positive for ATP1A1, ZO‐1, N‐cadherin, and vimentin	23	Lack of long‐term safety/tumorigenicity data; undefined conditioned medium causes GMP concerns	Chen et al., 2018 [103]
Biochemical induction methods	ESCs	Scalable, cryopreservable hESC‐CEC differentiation via efficient two‐step neural crest protocol	≥95% (positive for ATP1A1, ZO‐1, N‐cadherin, and hESCs < 0.001%)	24	No tumor/teratoma formation or systemic cell migration in vivo; xeno‐free, GMP‐compliant and safe in rabbit models.	Yu et al., 2023 [134]
	iPSCs	Sufficient functional CECs for clinical‐grade production	97.8% ATP1A1, 98.9% N‐cadherin, 99.4% mineralocorticoid receptor+, residual undifferentiated iPSCs <0.01%.	14	No tumorigenicity, cell residual or toxicity in 52‐week high‐dose assay; no 1‐year serious adverse events. Isolated EP300 exon22 deletion found with no adverse effects.	Hirayama et al., 2025 [29]
	MSCs	Stable differentiation from UC‐MSCs to CECs using GSK‐3β and dual ROCK inhibitors; Effective engraftment in rabbit models.	Positive for ATP1A1, ZO‐1, N‐cadherin, AQP1, COL8A1, COL8A2, CD166	16	No‐tumorigenic; Xeno‐free component used; No adverse events in 3 weeks rabbit study.	Ye et al., 2022 [47]
Mechanical methods	MSCs	Stable differentiation via DLT mechanotransduction, functionally mature and scaffold‐compatible.	High proportion of hexagonal CECs; Positive for ATP1A1, ZO‐1, PITX2, COL8A2.	16	No‐tumorigenic Physical induction only (no exogenous small molecules); Good biocompatibility in ex vivo cornea model.	Feiertag et al., 2020 [161]

Differentiation performance and clinical applicability also vary drastically across stem cell sources. ESCs possess strong differentiation potency, and ESCs‐derived CECs closely resemble native CECs in morphology and function; however, their clinical use is restricted by ethical concerns, nonspecific differentiation, uncontrolled proliferation, teratogenicity, and mandatory allogeneic transplantation carrying immune rejection [134]. iPSCs avoid ethical limitations and allow scalable ex vivo expansion. Autologous iPSCs represent an ideal candidate for personalized therapy, generating CECs with high differentiation efficiency and stable function. Nonetheless, challenges remain, including genomic instability, residual undifferentiated cells leading to teratoma risk, and mild autologous immunogenicity. High cost, inconsistent differentiation, and suboptimal purity further limit large‐scale application, even though recent clinical trials strongly support the translational potential of iPSC‐based corneal therapy [177]. MSCs represent ideal adult stem cells for autologous transplantation with no tumorigenic risk [178]. Their easy accessibility, high safety, and low immunogenicity support utility in both autologous and allogeneic settings. However, MSCs have inherent limitations in differentiation potential and proliferative capacity, and studies on MSCs‐derived CECs remain limited. Further efforts are needed to improve functional maturation and homogeneity, making this direction worthy of continued exploration.

Despite the immune‐privileged status of the eye, immunological and genetic barriers remain critical hurdles for translating ESCs‐ and iPSCs‐derived CECs into the clinic. It is essential to comprehensively evaluate the advantages, disadvantages, and translational obstacles of each stem cell source before clinical application. ESCs are limited to allogeneic use, MSCs are applicable for both autologous and allogeneic therapies, and iPSCs can be established from patient somatic cells. Notably, autologous or syngeneic stem cells are more effective than allogeneic cells in treating ocular diseases [179, 180]. To overcome immune barriers in allogeneic CECs therapy, emerging strategies including HLA‐matched cell banking, gene editing, and naive‐state reprogramming are being developed to improve safety and enable large‐scale clinical delivery [173].

Despite rapid progress in generating stem cell‐derived CECs, different cell sources (iPSCs, ESCs, and MSCs) rely on distinct induction systems, making it challenging to directly compare differentiation efficiency, purity, functional maturity, and therapeutic outcomes across protocols. To address this issue, future studies should establish standardized induction platforms, unified quality control indicators, and head‐to‐head comparison systems for CECs derived from different stem cell sources. High‐throughput transcriptomics, proteomics, single‐cell RNA sequencing, and phenotypic screening can be used to systematically compare gene expression profiles, functional states, cellular heterogeneity, and safety profiles. Establishing consistent evaluation criteria—including differentiation efficiency, purity, pump function, barrier integrity, long‐term survival, and tumorigenicity—will enable fair and reliable cross‐protocol comparisons. In addition, advanced cell culture systems and corneal organoid models will further facilitate the maturation and functional evaluation of stem cell‐derived CECs. Such studies will clarify the advantages and limitations of each stem cell source, guide the selection of optimal cell sources for clinical translation, and promote the development of more efficient, stable, and safe CECs differentiation systems. Ultimately, standardized comparative research may help accelerate the clinical application of stem cell‐based corneal endothelial regeneration.

## Author Contributions

**Ling Li**: literature collection, analytical summary, manuscript writing. **Yan Huang**: conceptual design of the review and manuscript revision. **Yuanyuan Wu and Qiuping Wang**: participation in manuscript writing. **Jing Ji and Lizhen Wang**: manuscript review and proofreading.

## Funding

This work was suppoted by the Beijing Municipal Natural Science Foundation (Grant Z240017) and the Fundamental Research Funds for the Central Universities.

## Disclosure

All authors have read and approved the final version of the manuscript. Jing Ji and Lizhen Wang have full access to all of the data in this study and take complete responsibility for the integrity of the data and the accuracy of the data analysis.

## Ethics Statement

The authors have nothing to report.

## Conflicts of Interest

The authors declare no conflicts of interest.

## Data Availability

Data sharing does not apply to this article as no datasets were generated or analyzed during the current study.
